# The Use of the FACE-Q Aesthetic: A Narrative Review

**DOI:** 10.1007/s00266-022-02974-9

**Published:** 2022-06-28

**Authors:** Maarten J. Ottenhof, Inge J. Veldhuizen, Lusanne J. v. Hensbergen, Louise L. Blankensteijn, Wichor Bramer, Berend vd Lei, Maarten M. Hoogbergen, René R. W. J. Hulst, Chris J. Sidey-Gibbons

**Affiliations:** 1 Division of Plastic and Reconstructive Surgery, Brigham and Women’s Hospital and Harvard Medical School, 75 Francis St, Boston, MA 02115 USA; 2 Department of Plastic and Reconstructive Surgery, Catharina Ziekenhuis, Eindhoven, The Netherlands; 3 Department of Plastic and Reconstructive Surgery, Maastricht University Medical Center, Maastricht, The Netherlands; 4 Patient-Reported Outcomes, Value & Experience (PROVE) Center, Department of Surgery, Brigham and Women’s Hospital, Boston, MA USA; 5 Medical Library, Erasmus MC, Erasmus University Medical Centre Rotterdam, Rotterdam, The Netherlands; 6 Department of Plastic Surgery, University and University Medical School of Groningen and Bey Bergman Clinics, Groningen, The Netherlands

## Abstract

**Introduction:**

In the past decade there has been an increasing interest in the field of patient-reported outcome measures (PROMs) which are now commonly used alongside traditional outcome measures, such as morbidity and mortality. Since the FACE-Q Aesthetic development in 2010, it has been widely used in clinical practice and research, measuring the quality of life and patient satisfaction. It quantifies the impact and change across different aspects of cosmetic facial surgery and minimally invasive treatments.

We review how researchers have utilized the FACE-Q Aesthetic module to date, and aim to understand better whether and how it has enhanced our understanding and practice of aesthetic facial procedures.

**Methods:**

We performed a systematic search of the literature. Publications that used the FACE-Q Aesthetic module to evaluate patient outcomes were included. Publications about the development of PROMs or modifications of the FACE-Q Aesthetic, translation or validation studies of the FACE-Q Aesthetic scales, papers not published in English, reviews, comments/discussions, or letters to the editor were excluded.

**Results:**

Our search produced 1189 different articles; 70 remained after applying in- and exclusion criteria. Significant findings and associations were further explored. The need for evidence-based patient-reported outcome caused a growing uptake of the FACE-Q Aesthetic in cosmetic surgery and dermatology an increasing amount of evidence concerning facelift surgery, botulinum toxin, rhinoplasty, soft tissue fillers, scar treatments, and experimental areas.

**Discussion:**

The FACE-Q Aesthetic has been used to contribute substantial evidence about the outcome from the patient perspective in cosmetic facial surgery and minimally invasive treatments. The FACE-Q Aesthetic holds great potential to improve quality of care and may fundamentally change the way we measure success in plastic surgery and dermatology.

**Level of Evidence III:**

This journal requires that authors assign a level of evidence to each article. For a full description of these Evidence-Based Medicine ratings, please refer to the Table of Contents or the online Instructions to Authors www.springer.com/00266.

**Supplementary Information:**

The online version contains supplementary material available at 10.1007/s00266-022-02974-9.

## Introduction

Facial Aesthetic procedures are the most performed procedures in plastic surgery [1]. The number of surgical and non-surgical procedures keeps rising steadily [1, 2].

To objectify improvement of (area-specific) appearance and overall health-related quality of life (HR-QOL) is often challenging. Patient-reported outcome measures (PROMs) have gained considerable traction in the past decades, adding value to patients, care providers, and the process of care [1]. The FACE-Q Aesthetic module was developed for facial esthetic procedures, and proved to be a reliable instrument to measure patient-reported outcomes following surgical and non-surgical facial rejuvenation [3].

The FACE-Q Aesthetic was developed using item response theory principles, which maximizes the applicability: The items within the scales are ranked clinically relevant and score a so-called underlying trait (e.g., ‘satisfaction with facial appearance’). Responses to the items are rated on a four-point Likert scale (strongly disagree strongly agree), summed, and transformed to a score from 0 to100 for interpretability. The scales are grouped in four categories: Health-related Quality of Life (10 scales), Appearance appraisal scales (24 scales), and Adverse Effect checklists (6 scales). Checklists functioning differently from scales. They cover a topic such as ‘complications’ but are not necessarily correlated (e.g., hematoma, infection) thus cannot be ranked on one scale since there is no underlying trait However, checklists can provide clinically important information, such as monitoring for post-treatment complications. Given the modular structure, researchers and clinicians can choose the scales suitable to their research question or clinical situation.

In this review, we set out to examine the use of the FACE-Q Aesthetic in the plastic surgery and dermatology research literature and how this has expanded the understanding of the facial esthetic practice.

## Methods

A literature search of studies using the FACE-Q Aesthetic scales as outcome measures was conducted by a trained medical librarian (WMB). Five search engines were queried: Embase via embase.com, MEDLINE ALL via Ovid, Cochrane CENTRAL registry of Trials via Wiley, with the limits set to articles published 2010 up to January 2021. Searches were specifically created aiming for PROMs (specific search terms in supplemental materials) [4].

**Fig. 1:**
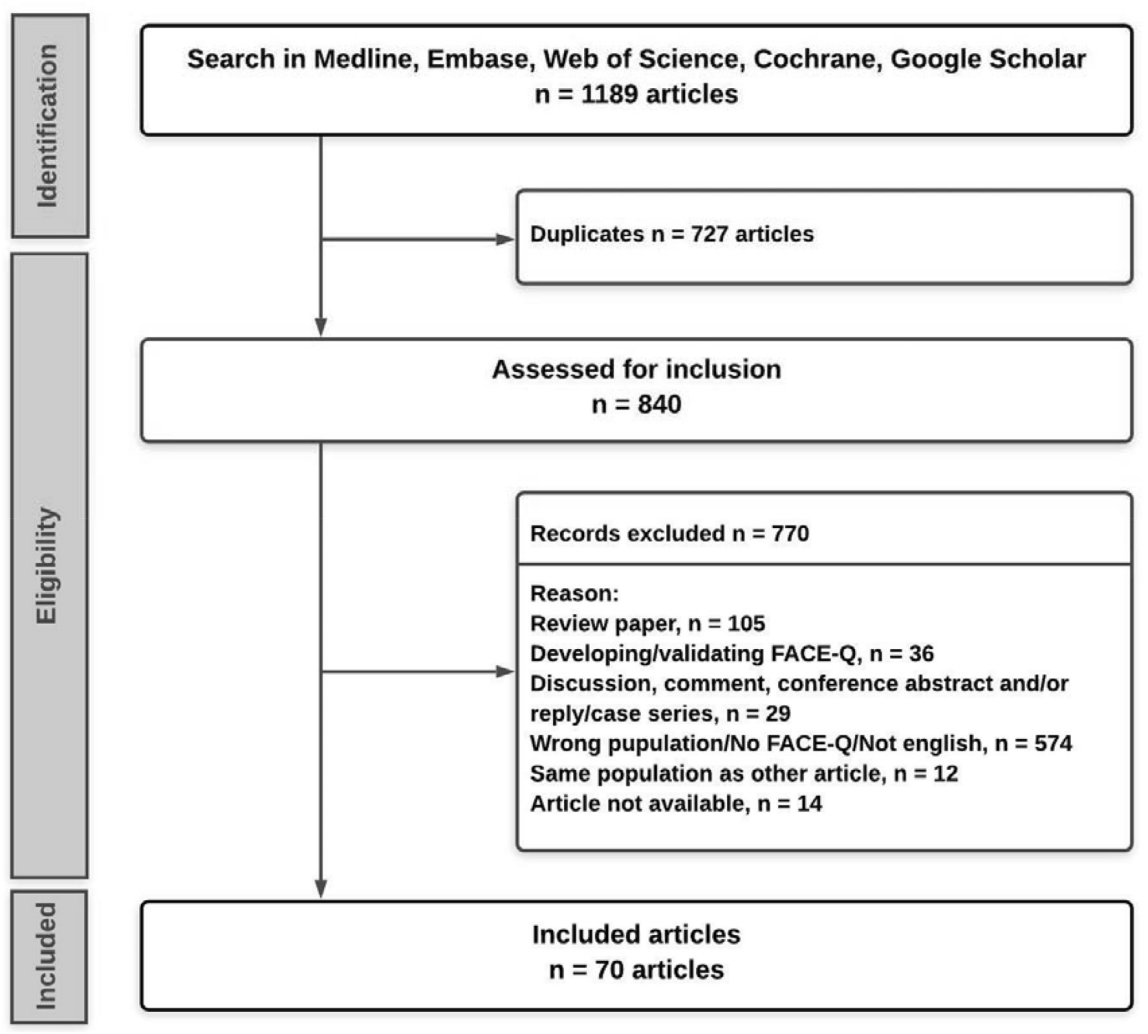
Flowchart of article selection

Articles were imported in Rayyan QCRI Review software and deduplicated using the method described by Bramer et al. [5]. Two authors (MO, IV) separately reviewed all the results by title and abstracts in a blinded fashion. All discrepancies were resolved through discussion or by a third author (Fig. 1).

Inclusion criteria consisted of using one or more FACE-Q Aesthetic scale to evaluate patient outcome, articles describing facial procedures, and primary research studies. Studies were excluded if they described the development of different PROMs, validated the FACE-Q Aesthetic in another language or culture, and if the format was either a conference abstract, (systematic) review, thesis, or a commentary (Table 1).

**Table 1:** In- and exclusion criteria

Inclusion criteria	Exclusion criteria
Using one or more domains of the FACE-Q esthetic to evaluate patient outcome	Developing and/or validating the FACE-Q or other PROM
Primary research study	Review
Facial procedures	Conference abstracts
	Thesis
	Commentaries
	Letters to the editor
	A modified version of FACE-Q Aesthetic
	Other languages than English

Full articles were reviewed. The following information was extracted: country of origin, sample size, FACE-Q Aesthetic scales, time of completion, and the level of evidence using Oxford Level of Evidence [6]. The time of completion of the FACE-Q Aesthetic was categorized in pre-treatment, short term (< 6 months post-treatment), and long term (> 6 months post-treatment). Scales Satisfaction with Decision, Satisfaction with Surgeon, Satisfaction with information, Satisfaction with office staff, Satisfaction with medical team, are not officially part of the Face-Q, we did include them in the review because a great number of articles did use them. It must be noted these scales are part of the Body-Q.

## Results

### Publications and Used Scales

Our search generated a total of 1189 results. After removing duplicates, a total of 840 unique publications remained. Following the abstract review, 166 articles remained for full-text analysis after applying exclusion criteria. A total of 70 articles from 13 different countries, published between 2012 and 2020, were included. Some metadata are displayed in Table 2.

**Table 2:** Characteristics of the included articles

Characteristics	Number
Total studies (*n*)	70
Number of patients	
Total	5746
Mean	86
Response rate (average with > FU point)	83.2
Before and after (no = 0, yes = 1)	35
Follow up (mean of max in months)	16.7
Follow up < 6months	51
Follow up > 6 months	32
Level of evidence	
Median	3
Mean	2.96

From the 70 articles, a total of 5746 patients completed one or more FACE-Q Aesthetic scales, with an average number of included patients of 86 per publication. The average response rate was 83%. Thirty–five studies (50%) administered the FACE-Q Aesthetic before and after treatment. The average follow-up time was 16.7 months (ranging from 1 day to 70 months). For all studies, the mean level of evidence (possible scores 1–5) was 2.96 (median 3), corresponding to a case-control design. The Botox and fillers category scored highest with a mean level of evidence of 2.3 (median 2), encompassing all, except one, clinical trials.

Table 3 shows all scales from the FACE-Q Aesthetic and the number of times it was utilized. Supplementary information: papers using the FACE-Q Aesthetic, is an additional table, too large to be in the main body of text. It shows all the references and the scales that were utilized.

**Table 3:** FACE-Q Aesthetic scales, number of times employed and papers in which they were used

FACE-Q scale	Total	References
Appearance appraisal scales		
Satisfaction with facial appearance overall	32	[7–40]
Satisfaction with skin	6	[22, 28, 36, 41–43]
Satisfaction with nose	11	[30, 32, 33, 38, 44–50]
Satisfaction with nostrils	7	[30, 37, 44–46, 48, 50]
Satisfaction with lips	5	[20, 36, 38, 51–55]
Satisfaction with forehead and eyebrows	0	–
Satisfaction with eyes	3	[56–58]
Satisfaction with eyelashes	0	–
Satisfaction with lower face and jawline	15	[10–15, 18, 20–22, 36, 37, 37, 55, 59, 60]
Satisfaction with chin	8	[13, 13, 15, 18, 31, 40, 59, 61]
Satisfaction with cheekbones	1	[15]
Satisfaction with cheeks	13	[9–15, 18, 28, 29, 36, 38, 60, 62]
Appraisal of lines–overall	4	[18, 22, 24, 63]
Appraisal of lines–between eyebrows	1	[64]
Appraisal of lines–forehead	1	[13]
Appraisal of lines–crow’s feet	0	–
Appraisal of lines–lips	3	[18, 53, 54]
Appraisal of lines–marionette	1	[18]
Appraisal of lines–nasolabial folds	11	[9, 10, 12–15, 18, 60, 65–68]
Appraisal of upper eyelids	0	–
Appraisal of lower eyelids	0	–
Appraisal of area under chin	6	[10, 11, 14, 40, 60, 69]
Appraisal of neck	6	[10–12, 14, 18, 60]
Total appearance appraisal scales	116	–
*Quality of life scales*		
Psychological function	30	[9–14, 16, 17, 20, 23, 24, 26–31, 33, 37, 38, 40, 55, 58–63, 70, 71]
Social function	26	[9–14, 16, 17, 20, 22, 27, 28, 30, 33, 37, 38, 48, 50, 55, 58, 60–62, 70–72]
Age appraisal	13	[9–11, 14, 16–18, 26–29, 35, 73]
Age appraisal VAS	12	[10–12, 14, 17, 23, 26, 27, 29, 35, 62, 64]
Expectations	2	[30, 64]
Appearance-related distress	5	[30, 33, 58, 74, 75]
Recovery-early life impact	15	[8, 9, 11–14, 38, 51–54, 60, 61, 69, 73]
Satisfaction with outcome	19	[9, 11–14, 16, 28, 29, 33, 38, 52, 60–62, 64, 72, 76–78]
Satisfaction with decision	15	[9, 11–16, 29, 38, 56, 60, 61, 64, 70, 72]
Satisfaction with surgeon	1	[61]
Satisfaction with information	0	–
Satisfaction with medical team	1	[61]
Satisfaction with office staff	0	–
Total quality of life scales	117	–
Adverse effects checklists		
Recovery–early symptoms	8	[13, 29, 52, 61, 62, 66, 69, 73]
Skin	3	[13, 29, 36]
Forehead, scalp, and eyebrows	0	–
Eyes	0	–
Nose	0	–
Cheeks, lower face and neck	4	[13, 37, 61, 79]
Lips	0	–
Total adverse effects checklists	15	–
Total (ALL)	243	**–**

The top 3 FACE-Q Aesthetic scales used, consisted of ‘Satisfaction with Facial Appearance Overall’ (30 articles), ‘Psychological Function’ (25 articles), and ‘Social Function’ (21 articles), respectively.

Of the scales measuring appearance, the ‘Appearance appraisal’ scales were used more than satisfaction scales (120 times total, 19 out of 23 scales used). Specific area scales that were most used are: ‘Satisfaction with Lower Face and Jawline’(12 studies), ‘Satisfaction with Cheeks’(12 studies) and ‘Satisfaction with Nose’ (10 studies), and ‘Appraisal of Nasolabial Folds’(10 studies).

The second most used group was the HR-QoL group, and relatively frequently used (7 scales were used 91 times). The scale most utilized in this group was the ‘Psychological Function’ scale, used in 25 articles (43%). The studies that did not include a HRQoL scale (21/58) employed only 1 (median) appearance appraisal scale, reflecting an overall less elaborate use of FACE-Q scales in those studies.

The Adverse Effects group (checklists) was the least utilized, 13 studies (22%). The most used checklist in this group was the ‘Recovery–Early Symptoms’ checklist, used in 8 articles (13.8%). Only two scales of the ‘Patient Experience of Care’ category were used. Some scales were not used at all.

### Facelift

A total of 10 articles comprising 563 patients used the FACE-Q Aesthetic for facelift procedures. 20 out of 40 independent FACE-Q Aesthetic scales were used. Three of these articles used the FACE-Q Aesthetic to evaluate patient-reported outcome before and after the facelift surgery. The average follow-up time was 27 months, with an average response rate of 72%. The median Oxford level of evidence score of these articles is 3. Most of these studies (70%) are retrospective and relatively small (average number of patients is 56 ranging from 13 to 124).

The most used scales targeting facial features were “Satisfaction with Facial Appearance Overall (8)”, “Satisfaction with Cheeks (8)”, “Satisfaction with Lower Face and Jawline (6)”, “Appraisal of Nasolabial Folds (6)”. The majority of the articles also measured “Psychological Function (7)”, “Social Function (7)”, “Satisfaction with Outcome (7),” and “Satisfaction with Decision (7)”.

The articles in this review used 20 out of 40 independent FACE-Q scales. Quantifying overall- and area-specific satisfaction to help understand the impact of facelift surgery.

Results show durability (1 year) [8, 62] and high satisfaction with facial appearance and psychological function for both surgical or minimally invasive facelift [8, 11, 14, 15, 62, 80]. Sinno et al. found that patients felt they appeared 2.5–7 years younger [14] .The use of area-specific FACE-Q scales showed significant improvement in multiple facial regions [9–11, 62, 80]. For example, using an alternative incision in bald facelift patients, Pascali et al. [12] found high satisfaction for the various critical areas examined, including scars. A facelift combined with blepharoplasty was significantly associated with a more remarkable improvement in satisfaction than a facelift without a blepharoplasty [10].

### Rhinoplasty

A total of 10 articles comprising 937 patients (average of 94) used the FACE-Q Aesthetic to evaluate rhinoplasty procedures. The average follow-up item was 15 months, with an average response rate of 85%. Seventy percent collected data pre- and post-rhinoplasty. The average level of evidence for these articles is 2.7 (median 3). The “Satisfaction with Nose” scale was used in all ten articles. Followed by “Satisfaction with Nostrils” (5) and “Satisfaction with Facial Appearance Overall” (4), “Psychological Function” (4), and “Social Function” (5). Interestingly, the scales used to evaluate satisfaction with outcome or decision were used only once, compared to the facelift articles.

In rhinoplasty patients, factors such as age, race and income showed to be predicting success.

Studies showed an overall increase in quality of life, satisfaction with facial appearance, social function, and psychological function after rhinoplasty [30–32, 44, 48, 49]. Interestingly, not all groups benefited equally from a rhinoplasty. Two articles found being young, being of the Caucasian race, and having a high income associated with improved FACE-Q scores after rhinoplasty. Men reported improved satisfaction with facial appearance after rhinoplasty, but this did not result in a higher HRQoL than women [31, 44]. Also, Barone et al. found that older patients (> 65 years old) tend to be more focused on the tip of the nose [44].

### Injectables

A total of 23 articles, including 2292 patients (average of 97), used the FACE-Q Aesthetic to evaluate injectable procedures. Of these articles, 12 had before and after measurements, the average follow-up time was 6.3 months. The response rate was 91% on average.

The most used appearance scale is “Satisfaction with Facial Appearance Overall” (9), after which “Satisfaction with Lips/Lower Face and Jawline (both 4) and “Appraisal of Nasolabial Folds” (also 4). The most used HR-QOL scale was “Psychological Function” (6), after which “Early Life Impact of Treatment,” “Age Appraisal,” and “Satisfaction with Outcome” were four times utilized. This category’s diverse character is reflected in the use of scales; not one scale is used by the majority of articles. For minimally invasive techniques, the FACE-Q was used mainly area-specific and proved to detect even small changes in satisfaction.

We found two studies measuring the effect of solely botulinum toxin treatment using the FACE-Q Aesthetic. These small-scale studies (n = 15 and 50 respectively) show significantly increased satisfaction on all FACE-Q domains after botulinum toxin treatment [18, 64]. Also, when using multimodal minimally invasive approaches to facial esthetic treatment, the outcome of perceived age after treatment seems to increase significantly [17, 18] and helped to guide daily practice [16, 17, 22, 24]—especially insights in selecting and combining treatment modalities. When performed by residents, nonsurgical facial rejuvenation procedures can improve patients’ quality of life and provide high satisfaction without compromising safety and enable constructive feedback. [16, 19] Fillers represent a valuable adjunct to surgical procedures for improving facial esthetics after injury and, consequently, patients’ quality of life affected by facial trauma. [24] Several studies used the FACE-Q to evaluate soft tissue fillers’ safety and effectiveness for peri–oral enhancement or midface contour deficiencies, a new type of filler for nasolabial folds [34], or jaw restoration [81]. These controlled trials [52, 53, 65, 66, 81] and prospective cohort studies [54, 78] demonstrated a significant improvement in satisfaction with facial appearance and showed the FACE-Q’s ability to detect small changes in satisfaction. Doyle et al. [57] found no significant difference between patients with silicone oil in situ and those with a phthisical eye (shrunken eye), providing valuable data for the shared decision-making process when considering this procedure, given its attached therapeutic, logistical, and financial implications. Bertossi et al. [75] Substantiated their algorithmic approach for facial fillers with FACE-Q scores.

## Discussion

Since the introduction of the FACE-Q Aesthetic in 2010, it has been used to study facial surgery outcomes and helped to gain knowledge of the patient perspective and further deepen the understanding of what makes (non)-surgical esthetic procedures successful. The FACE-Q Aesthetic responses have shed light on evidence of patient satisfaction with (combinations) of different (non)-surgical techniques that would otherwise have stayed obscured. In facelifts durable results were shown, techniques were refined, patients felt they appeared 2.5–7 years younger and specific techniques such as scar placement in bald patients were investigated. In rhinoplasty patients, factors such as age, race and income showed to be predicting success. For minimally invasive techniques, the FACE-Q was used mainly area-specific and proved to detect even small changes in satisfaction.

**Fig. 2:**
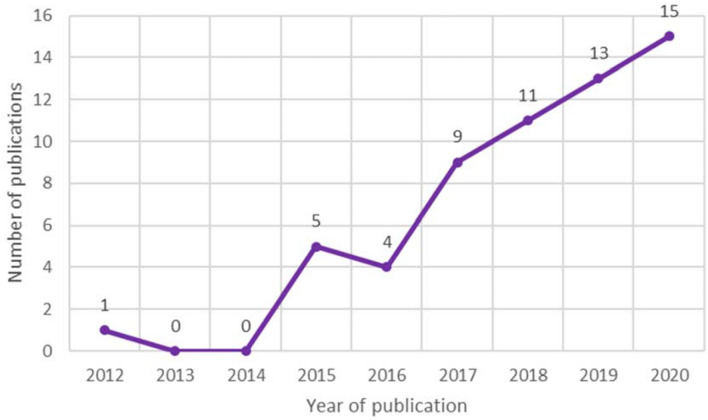
Number of articles using the FACE-Q Aesthetic by year

Since most cosmetic procedures are facial procedures, the impact of the FACE-Q could be substantial. The FACE-Q provides a standardized method to evaluate outcome, additionally providing the opportunity to monitor (rare) complications and adverse effects, as reported in Guarro et al.[82]. Since its inception, FACE-Q’s uptake has been increasing and more than tripled in the last three years (Fig. 2).

Some scales within the Appearance group were not used in published research. These scales measure appearance of eyelashes, crow’s feet, and lower/upper eyelid scales. The latter we found surprising because eyelid surgery is in the top 3 cosmetic surgical procedures.

Most studies used only a tiny subset of scales. It shows the versatility of the thirty–nine independently functioning scales and checklists but, in many cases, also leaves untapped potential since the FACE-Q comprises multiple scales, targeting different facial areas of interest, broadening its applicability. We expect that using the different FACE-Q Aesthetic scales will continue to increase since the importance of PROMS has been rapidly accepted by policymakers, clinicians, and researchers. Below we consider three main areas of practice and expand upon this review’s findings to shed light on where the FACE-Q Aesthetic has (and can) been used to understand the evidence-based, patient-centered surgical practice better.

### Facelift, a Warm Welcome for PROMS

The potential of face lifting is unmatched in its ability to rejuvenate a sagging facial shape, affecting almost every area of the face. Numerous variations in the technique of facelift surgery have been described from skin excision only, to deep plane facelifts, to the current superficial musculoaponeurotic system operations. Instead of one technique being superior to another, a competent surgeon is likely to produce satisfactory results with different techniques when patients are appropriately selected. Although numerous studies have tried to compare outcomes produced by different techniques, high-quality data are scarce [83]. The FACE-Q adds a valuable perspective on the effectiveness of facelift procedures alongside traditional outcome measures such as morbidity and may provide the means to incorporate patient perspective and individualize our daily practice's algorithmic approach. For example, one study showed their artificial intelligence model to correctly estimate patients’ age reduction after different facelift techniques [60].

### Rhinoplasty, Patient Characteristics and Prediction

Rhinoplasty is one of the most common operations performed–in 2020, well over 200.000 in the US alone [84].

It is among the most complex procedures in plastic surgery, and patients often have high expectations. Quantifying patient satisfaction pre- and post-rhinoplasty using FACE-Q scales (per area a specific scale could be added to obtain more specific insight alongside the more general scales) could help identify factors that hold a predictive value for success. And thus, it allows surgeons to determine in which areas and among which groups they are producing surgical success and where and among which groups they fail to do so. Moreover, it enables the surgeon to effectively identify specific anatomical areas of interest, patient expectations and, tailor treatment strategies.

We found age, income, and gender were found to be predictive of FACE-Q Aesthetic scores. Although it is beyond this review’s scope to speculate about the reasons behind the identified factors, other baseline characteristics such as disturbed body image, cause, history, and type of underlying deformity should be appreciated when interpreting outcomes [85].

### Injectables (Botox and Fillers), FACE-Q Added to the Armamentarium

By far, botulinum toxin treatment is the most commonly performed facial enhancement method in the USA, followed by soft tissue fillers [1]. Together they generate close to $2 billion annually. We found that the use of Botox was associated with an increase in FACE-Q Aesthetic scores for all area specific scales, demonstrating effectiveness of Botox treatment and sensitivity of the FACE-Q Aesthetic as an instrument. Combining Botox and fillers in the aging face seems logical since skin quality and fat distribution change. The combined use lowered perceived age in patients. Also, after reconstructing traumatic wounds, fillers improved facial esthetics. These results seem to show great potential for the use of injectables in reconstructive surgery.

### Difference in Score, When is it Important?

What are we to conclude if we find a difference in, e.g., four points FACE-Q score? Is the treatment effect large, warranting a widespread change in treatment strategies, or is it immaterial, suggesting no added value of the treatment? For example, the FACE-Q was used alongside 3D imagery in different studies to maximize the interpretability of their results: submental fat volume changes after deoxycholic acid injection [69, 77] nose morphometry after reconstruction [45], changes in facial appearance after treatment with sex hormones [25], and lower jaw contouring with titanium implants [37]. But how much change in score would mean a clinically relevant difference?

The minimally important difference (MID) provides a measure of the slightest change in the PRO of interest that patients perceive as important, leading the patient or clinician to consider a change in management [86]. Weinkle et al. [17] noticed a similar increase in FACE-Q score for their multimodal approach of non-surgical esthetic procedures to some studies evaluating surgical procedures. However, although a MID may help compare results across studies, care must be taken in applying the MID as it may differ for different situations as patient populations may vary.

There are two primary approaches for estimating a MID: distribution-based and anchor-based methods [87].

Heuristically—using the distribution-based method—one could take half the standard deviation of the mean score [86]. Using this method, in 2017, Klassen et al. found a MID for ‘Satisfaction with facial appearance’ of 7.0 for surgical and 7.1 for non-surgical procedures. Weitzman used the mean baseline standard deviation to calculate the MID for three scales: ‘Satisfaction With Nose’ scale (11.0); ‘Satisfaction With Nostrils’ (13.6); ‘Social Functioning’ (10.2). The anchor-based approach to calculating a MID compares changes in scores with an ‘anchor’ as a reference. Hall et al. looked at whether patients were still supportive of their decision to undergo treatment after peri–oral fillers [56].That binary question could potentially serve as an anchor for patients that seek peri–oral fillers. Although there are no accepted standards for appraising MID estimates’ credibility. [88], we suggest that future FACE-Q publications do calculate a MID, providing an intuitive measure for decision-makers.

### Where to Go From Here?

As described in this review, the FACE-Q Aesthetic is increasingly used to determine the impact of different (combinations of) facial cosmetic procedures.

As plastic surgeons and dermatologists are eager to improve their practice using evidence-based medicine [89], with increase in volume and different types of facial esthetic procedures available, it is expedient to incorporate patient outcome measures routinely. FACE-Q implementation could be facilitated by using electronic data capture (EDC) software. Through the senior author, ready-to-use EDC formats can be obtained. Also, Computerized Adaptive Testing (CAT) could be applied- as it was developed using modern test theory (Rasch) -to reduce the burden for the patient to a minimum. Depending on the patients’ responses the computer selects the next item from the scale that provides the most information. It shortens the questionnaires during the assessment, while retaining accuracy [90, 91]. Data collected could be used in predictive models, helping doctors to identify patients who are highly likely to benefit from surgery and guide those unlikely to benefit from other treatment options [92].

The FACE-Q Aesthetic module and our review of the literature have several limitations. This review was not systematic and could have some omissions. However, we did not set out to conduct a systematic review. We did not have a specific question to answer; we wanted to provide healthcare providers with an overview of the published surgical research. Also, we did not include non-peer-reviewed studies, or those from conferences, although they might have contributed meaningful clinical data. Longitudinal research’s validity and reliability are highly dependent on the recruitment and retention of representative samples. The reported response rates of several references were marginal, impacting the studies’ reliability. Although the largest series in facelift started with 200 patients, it reported a response rate of only 38% [15]. Researchers may need practical and methodological support when asked to assist in collecting FACE-Q data to minimize bias. We should be cautious interpreting PROM results when comparing different techniques as outcomes do not only reflect procedures’ technical success. This makes it essential to combine PROM results with surgical or technical outcomes.

The discrepancy between the number of esthetic facial procedures performed and the FACE-Q’s use creates an opportunity to expand the understanding of treatment possibilities and patient satisfaction and improve our standard of care. Therefore, we encourage surgeons and dermatologists to introduce patient-reported outcome measurements into their esthetic practice in the future.

## Conclusions

The FACE-Q is a PROM that allows both researchers and clinicians to answer essential questions on patient satisfaction, HR-QOL and adverse effects. Its modular structure with multiple scales enables researchers and clinicians to comprehensively answer clinical questions specific to surgical and nonsurgical facial interventions. The standardized scoring methodology is simple to use and permits comparisons between studies. FACE-Q’s uptake in research has markedly helped to amass knowledge of the patient perspective and increase the understanding of what makes esthetic procedures successful*.* While the use of FACE-Q has provided numerous valuable insights so far, the increased interest in PROs guarantees its continued use and ability to harbor innovations and standards of care.

## Supplementary Information

Below is the link to the electronic supplementary material.

Supplementary file1 (DOCX 14 KB)

Supplementary file2 (CSV 89 KB)
