# Supplementary material for: The Use of the FACE-Q Aesthetic: A Narrative Review
Source: Aesthetic Plast Surg. 2022 Jun 28;46(6):2769–80. doi: 10.1007/s00266-022-02974-9 (PMC9729314; doi:10.1007/s00266-022-02974-9)
Supplement: Supplementary file 1 — Supplementary file1 (DOCX 14 KB) [file 266_2022_2974_MOESM1_ESM.docx]

**SUPPLEMENTAL MATERIALS**

**Supplement 1**: SEARCH TERMS PER SEARCH ENGINE

***embase.com 254***

*(('wrinkle'/exp OR 'skin atrophy'/de OR 'facial aging'/exp OR (wrinkl* OR rhytid* OR ((skin OR facial OR cheek* OR chin OR nose OR nasal OR forehead OR Mouth OR Nasolabial OR temple OR crow-s-feet OR cro*ws-feet OR canthal OR glabellar) NEAR/3 (atroph* OR Hollowing OR Sagging OR Redundanc* OR Drooping OR crease OR lines)) OR (Tear-Through NEAR/3 deform*)):ab,ti) AND ('therapy'/exp OR 'surgery'/exp OR 'therapy':lnk OR 'surgery':lnk OR (therap* OR treat* OR surg* OR nonsurg* OR operati* OR nonoperati*):ab,ti)) OR ('rejuvenation'/exp OR 'rhytidoplasty'/de OR 'injectable implant'/exp OR 'chemexfoliation'/de OR 'skin abrasion'/de OR 'low level laser therapy'/de OR (Rejuvenat* OR ((brow OR Forehead OR cheek OR chin OR Eyelid OR face OR facial OR neck OR nose OR skin) NEAR/3 (lift OR Surgery OR Reduction OR Augmentation OR Enhancement OR implant* )) OR (Buccal-Fat NEAR/3 Removal ) OR Facelift* OR rhytidoplast* OR Filler* OR chemexfoliat* OR Chemical-Peel OR (laser NEAR/3 (therap* OR Resurfac*)) OR Microdermabras* OR (Skin NEXT/1 Resurfaci*) ):ab,ti) AND ('patient-reported outcome'/exp OR ((patient* NEAR/3 report* NEAR/3 outcome*) OR proms OR prom):ab,ti) OR ('face q' OR faceq):ab,ti

**Medline Ovid 173**

((Skin Aging/ OR Skin Aging/ OR (wrinkl* OR rhytid* OR ((skin OR facial OR cheek* OR chin OR nose OR nasal OR forehead OR Mouth OR Nasolabial OR temple OR crow-s-feet OR crows-feet OR canthal OR glabellar) ADJ3 (atroph* OR Hollowing OR Sagging OR Redundanc* OR Drooping OR crease OR lines)) OR (Tear-Through ADJ3 deform*)).ab,ti.) AND (exp therapeutics/ OR exp Surgical Procedures, Operative/ OR therapy.fs. OR surgery.fs. OR (therap* OR treat* OR surg* OR nonsurg* OR operati* OR nonoperati*).ab,ti.)) OR (Rejuvenation/ OR Rhytidoplasty/ OR Plasma Skin Regeneration/ OR Chemexfoliation/ OR Dermabrasion/ OR Low-Level Light Therapy/ OR (Rejuvenat* OR ((brow OR Forehead OR cheek OR chin OR Eyelid OR face OR facial OR neck OR nose OR skin) ADJ3 (lift OR Surgery OR Reduction OR Augmentation OR Enhancement OR implant* )) OR (Buccal-Fat ADJ3 Removal ) OR Facelift* OR rhytidoplast* OR Filler* OR chemexfoliat* OR Chemical-Peel OR (laser ADJ3 (therap* OR Resurfac*)) OR Microdermabras* OR (Skin ADJ Resurfaci*) ).ab,ti.) AND (Patient Reported Outcome Measures/ OR ((patient* ADJ3 report* ADJ3 outcome*) OR proms OR prom).ab,ti.) OR (face q OR faceq).ab,ti.

**Web of Science 142**

TS=(((((wrinkl* OR rhytid* OR ((skin OR facial OR cheek* OR chin OR nose OR nasal OR forehead OR Mouth OR Nasolabial OR temple OR crow-s-feet OR crows-feet OR canthal OR glabellar) NEAR/2 (atroph* OR Hollowing OR Sagging OR Redundanc* OR Drooping OR crease OR lines)) OR (Tear-Through NEAR/2 deform*))) AND ((therap* OR treat* OR surg* OR nonsurg* OR operati* OR nonoperati*))) OR ((Rejuvenat* OR ((brow OR Forehead OR cheek OR chin OR Eyelid OR face OR facial OR neck OR nose OR skin) NEAR/2 (lift OR Surgery OR Reduction OR Augmentation OR Enhancement OR implant* )) OR (Buccal-Fat NEAR/2 Removal ) OR Facelift* OR rhytidoplast* OR Filler* OR chemexfoliat* OR Chemical-Peel OR (laser NEAR/2 (therap* OR Resurfac*)) OR Microdermabras* OR (Skin NEAR/1 Resurfaci*) ))) AND (((patient* NEAR/2 report* NEAR/2 outcome*) OR proms OR prom)) OR ("face q" OR faceq)) AND DT=(article) AND LA=(english)

**Cochrane CENTRAL 125**

((((wrinkl* OR rhytid* OR ((skin OR facial OR cheek* OR chin OR nose OR nasal OR forehead OR Mouth OR Nasolabial OR temple OR crow-s-feet OR crows-feet OR canthal OR glabellar) NEAR/3 (atroph* OR Hollowing OR Sagging OR Redundanc* OR Drooping OR crease OR lines)) OR (Tear-Through NEAR/3 deform*)):ab,ti) AND ((therap* OR treat* OR surg* OR nonsurg* OR operati* OR nonoperati*):ab,ti)) OR ((Rejuvenat* OR ((brow OR Forehead OR cheek OR chin OR Eyelid OR face OR facial OR neck OR nose OR skin) NEAR/3 (lift OR Surgery OR Reduction OR Augmentation OR Enhancement OR implant* )) OR (Buccal-Fat NEAR/3 Removal ) OR Facelift* OR rhytidoplast* OR Filler* OR chemexfoliat* OR Chemical-Peel OR (laser NEAR/3 (therap* OR Resurfac*)) OR Microdermabras* OR (Skin NEXT/1 Resurfaci*) ):ab,ti)) AND (((patient* NEAR/3 report* NEAR/3 outcome*) OR proms OR prom):ab,ti) OR ('face q' OR faceq):ab,ti

**Google scholar**

Wrinkles|wrinkling|"skin|facial atrophy|Hollowing|Sagging|aging" Rejuvenation|"face|facial lift"|Reduction|Augmentation|Enhancement|"Buccal-Fat Removal"|Facelift|rhytidoplasty|Filler|"laser therapy" "patient reported outcome|outcomes"

**Supplement 2**: FACE-Q references, which scales were used table in excel.
